# Supplementary material for: Learning curve and surgical outcome of robotic assisted colorectal surgery with ERAS program
Source: Sci Rep. 2022 Nov 29;12:20566. doi: 10.1038/s41598-022-24665-w (PMC9709162; doi:10.1038/s41598-022-24665-w)
Supplement: Supplementary file 1 — Supplementary Information 1. [file 41598_2022_24665_MOESM1_ESM.zip › LSC Raw data-Ñ[▒K/131/N_ñw╝╨░O▒Kñσ.pdf]

W95 / W95

臺中榮民總醫院
手術室護理記錄

[redacted]

基本資料

- 室別： 二醫手術室-31室，手術科別： CRS，手術類別： 常規，日期： 2021/08/11
- 進入時間： 0755 ； 麻醉開始時間： 0800 ； Time Out時間： 0853 ； 劃刀時間： 0854 ； 手術結束時間： 1110 送出時間： 1130 ；
- 麻醉方式： GE
  診斷：A Diverticulosis
  手術名稱：Laparoscopic right colectomy and anastomosis
  手術局部用藥：NIL
  術前預防性抗生素：
  - 抗生素名稱一： Veterin 1GM IVD/由麻醉護理執行
  - 時間： 0830
  - 上次簽章: 彭馨慧

  ，追加預防性抗生素：
- 手術醫師： 林俊余
- 麻醉醫師： VS 張思玲
-
  - 刷手護士A ： 林采儀，時間： 0755
  - 流動護士A ： 彭馨慧，時間： 0755

皮膚狀況

A-V Shunt： 無
術前： 完整
記錄者： 彭馨慧
術後： 不完整
- 2.瘀傷

部位(術後)：

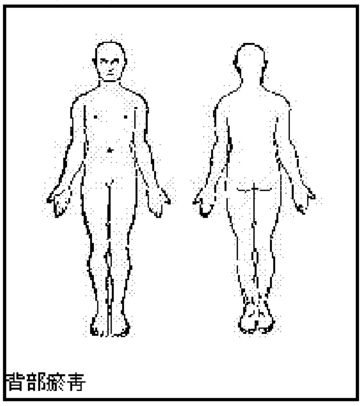

記錄者： 彭馨慧

手術準備

手術臥式 單一手術臥式

-
  - 手術臥式： 仰臥
  - 使用附件： 溫毯 38 ℃，頭圈
  - 皮膚準備： 10%Aq Betadine，Hibiscrub，2% Chlorhexidine
  - 電燒： 使用電燒: 電極片位置： 其它： 使用床上迴路電極墊，記錄者： 上次簽章: 彭馨慧

止血帶T： 無

植入物

- 植入物名稱/號碼/數量/部位：Ethicon ligaclip/20釘/pelvic

- Hyalobarrigel/pelvic/壹
  Ethicon EC60A/三釘/colon

, 記錄者： 上次簽章: 彭馨慧

標本送檢

- 細菌培養 無
- 細胞學 無
- 冰凍切片 無
- 病理標本
  組織名稱一： A-colon
- 截肢 無

標本交付 標本： 壹 袋 ， 標本： 零 盒 ， 標本： 零 瓶 ， 細胞學： 零 管 ， Culture Tube： 零 管 ， 被交付醫師： Fellow 陳映菁 ，

- 記錄者： 上次簽章: 彭馨慧
- 時間： 1050

傷口術後敷料

傷口敷料： 傷口敷料： 無

- 請於圖上標示部位：

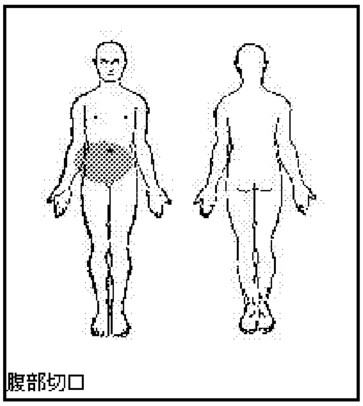

- 傷口敷料： menpor覆蓋

導管

導管	天數	導管資訊	插入日期	插入時間	更換日期	拔除日期	拔除時間	拔除原因	預計拔除/更換	預計拔除/更換日期
Penrose Drain(1)	1	號碼:12 mm.; 部位:肝下;	2021/08/11	1040						
A-line (1)	1	號碼:20號 ; 部位:右手;	2021/08/11	0835						
Foley(1)	1	號碼:16 Fr.; 部位:尿道;	2021/08/11	0825						
Sump NG Tube(1)	1	號碼:16 Fr.; 部位:胃;	2021/08/11	0830						

用物計數

- 手術中出血量： 少量 c.c.
- 手術中尿量： 300 c.c.
-
  - 其它： 無

用物計數： 多切口同時縫合

- 1.器械： 正確 ，2.縫針： 正確 ，3.刀片 ： 正確 ，4.紗布： 正確 ，5.腹部墊： 未使用 ，6.紗球： 未使用 ， 7.PEANUTS ： 未使用 ，8.紗條： 未使用 ，9.絲帶： 未使用 ，10.針頭： 未使用 ，11.Vessel loop： 未使用 ， 12.Boots： 未使用 ，13.Jelco： 未使用 ，14.棉條： 未使用 ，
  - 第一次：
    - 刷手護士 ： 林采儀
    - 流動護士： 上次簽章: 彭馨慧
    - 時間： 1020

,

- 第二次：
  - 刷手護士 ： 林采儀
  - 流動護士： 上次簽章: 彭馨慧
  - 時間： 1045

,

- 第三次：
  - 刷手護士 ： 林采儀
  - 流動護士： 上次簽章: 彭馨慧
  - 時間： 1100

備註

備註： NIL

- 記錄者： 上次簽章: 彭馨慧
- 時間： 1100

填寫護理人員 彭馨慧

正式摘要

W95 / W95

doctorRank:B
